# Supplementary material for: Development of a checklist of standard items for processing individual participant data from randomised trials for meta-analyses: Protocol for a modified e-Delphi study
Source: PLoS One. 2022 Oct 11;17(10):e0275893. doi: 10.1371/journal.pone.0275893 (PMC9553056; doi:10.1371/journal.pone.0275893)
Supplement: Attachment — Submitted filename: Response to Reviewers.docx [file pone.0275893.s002.docx]

**Point-by-point responses:**

**Journal Requirements**

When submitting your revision, we need you to address these additional requirements.

1. Please ensure that your manuscript meets PLOS ONE's style requirements, including those for file naming. The PLOS ONE style templates can be found at

<https://journals.plos.org/plosone/s/file?id=wjVg/PLOSOne_formatting_sample_main_body.pdf> and

<https://journals.plos.org/plosone/s/file?id=ba62/PLOSOne_formatting_sample_title_authors_affiliations.pdf>

**Response:** Thank you for sharing these links. The manuscript has been checked to ensure it meets all of PLOS ONE’s style requirements.

2. Thank you for stating the following in the Competing Interests section:

"I have read the journal's policy and the authors of this manuscript have the following competing interests: KEH receives research funding support via two scholarships administered by the University of Sydney (Postgraduate Research Supplementary Scholarship in Methods Development (SC3504), and Research Training Program Stipend (SC3227)). ALS is co-convenor and KEH & ACW are associate convenors of the Cochrane Prospective Meta-analysis Methods Group. MJP is recipient of the Australian Research Council Discovery Early Career Researcher Award (DE200101618), co-convenor of the Cochrane Bias Methods Group, and President of the Association for Interdisciplinary Meta-research and Open Science. RW is recipient of a National Health and Medical Research Council Investigator Grant. VB is supported by an Alfred Deakin Postdoctoral Research Fellowship. MC is co-convenor (unpaid) of the Cochrane Individual Participant Data Meta-analysis Methods Group; LHMR is coordinator of this group; KEH, PJG, BWM, MC and ALS are members. LHMR is supported by the UK Medical Research Council (https://mrc.ukri.org/) Grant number: MC_UU_00004/06. ALS is recipient of a National Health and Medical Research Council Investigator Grant. BWM is recipient of a National Health and Medical Research Council Investigator grant (GNT1176437), reports consultancy for ObsEva at an hourly rate, reports consultancy for Merck Merck KGaA at an hourly rate and received travel support from Merck Merck KGaA."

Please confirm that this does not alter your adherence to all PLOS ONE policies on sharing data and materials, by including the following statement: "This does not alter our adherence to PLOS ONE policies on sharing data and materials.” (as detailed online in our guide for authors http://journals.plos.org/plosone/s/competing-interests). If there are restrictions on sharing of data and/or materials, please state these. Please note that we cannot proceed with consideration of your article until this information has been declared.

Please include your updated Competing Interests statement in your cover letter; we will change the online submission form on your behalf.

**Response:** I confirm that this does not alter our adherence to all PLOS ONE policies, and have updated the Competing Interests section as required:

"I have read the journal's policy and the authors of this manuscript have the following competing interests: KEH receives research funding support via two scholarships administered by the University of Sydney (Postgraduate Research Supplementary Scholarship in Methods Development (SC3504), and Research Training Program Stipend (SC3227)). ALS is co-convenor and KEH & ACW are associate convenors of the Cochrane Prospective Meta-analysis Methods Group. MJP is recipient of the Australian Research Council Discovery Early Career Researcher Award (DE200101618), co-convenor of the Cochrane Bias Methods Group, and President of the Association for Interdisciplinary Meta-research and Open Science. RW is recipient of a National Health and Medical Research Council Investigator Grant. VB is supported by an Alfred Deakin Postdoctoral Research Fellowship. MC is co-convenor (unpaid) of the Cochrane Individual Participant Data Meta-analysis Methods Group; LHMR is coordinator of this group; KEH, PJG, BWM, MC and ALS are members. LHMR is supported by the UK Medical Research Council (https://mrc.ukri.org/) Grant number: MC_UU_00004/06. ALS is recipient of a National Health and Medical Research Council Investigator Grant. BWM is recipient of a National Health and Medical Research Council Investigator grant (GNT1176437), reports consultancy for ObsEva at an hourly rate, reports consultancy for Merck Merck KGaA at an hourly rate and received travel support from Merck Merck KGaA. This does not alter our adherence to PLOS ONE policies on sharing data and materials."

3. Your ethics statement should only appear in the Methods section of your manuscript. If your ethics statement is written in any section besides the Methods, please move it to the Methods section and delete it from any other section. Please ensure that your ethics statement is included in your manuscript, as the ethics statement entered into the online submission form will not be published alongside your manuscript.

**Response:** As requested, the ethics statement has been moved to the Methods section of our manuscript.

4. Please include captions for your Supporting Information files at the end of your manuscript, and update any in-text citations to match accordingly. Please see our Supporting Information guidelines for more information: http://journals.plos.org/plosone/s/supporting-information.

**Response:** A caption for the supporting information file (PRISMA checklist) has been added to the end of our manuscript. It is not cited in-text.

5. Please review your reference list to ensure that it is complete and correct. If you have cited papers that have been retracted, please include the rationale for doing so in the manuscript text, or remove these references and replace them with relevant current references. Any changes to the reference list should be mentioned in the rebuttal letter that accompanies your revised manuscript. If you need to cite a retracted article, indicate the article’s retracted status in the References list and also include a citation and full reference for the retraction notice.

**Response:** The reference list has been reviewed and, to our knowledge, it is complete and correct. No changes have been made and no retracted articles have been cited.

**Reviewer’s comments to the Author**

Reviewer #1: I thank the authors for contributing to the field of research quality and integrity. The authors highlighted the importance of the PRIME-IPD framework but also its limitation and they propose to develop a Delphi-informed checklist and a companion R-markdown program (strength of the project) to guide routine data quality checking and cleaning for IPD-MA of randomised trials. Overall, the manuscript is well-writing and documented.

**Response:** Many thanks for this positive feedback.

I provided some comments, mostly related to phase 1-scoping review, to help improve the manuscript.

-Major

Page 6. Please provide the direct link of OSF to the registered protocol instead of the general OSF website so that the reviewers and readers can access the protocol.

**Response:** Thank you for this suggestion. The direct link to the publicly available protocol has been added to the manuscript as follows:

“A full protocol for this review, covering each relevant item of the Preferred Reporting Items for Systematic Reviews and Meta-Analyses Extension for Scoping Reviews (PRISMA-ScR) (18), has been preregistered on the Open Science Framework (OSF, <https://osf.io/g2unf/>).” (p.7)

-Minor

Page 7. The following statement “We will use a standardised data extraction form and check any available protocol publications, results publications and/or supplementary materials (e.g., statistical analysis plans, data management plans, PROSPERO registration records) for each included IPD-MA) is a bit confusing by combining the use of a standardised data extraction form and the process of looking for available protocol publications, etc. in the same sentence. For example, I do not know if the search of the PROSPERO database is part of a structured grey literature search or not. I was not able to get the OSF-registered protocol.

**Response:** Thank you for this comment. We have re-worded relevant sections of this paragraph for clarity:

“We will include systematic reviews with IPD-MA of randomised trials on intervention effects published in English. These have previously been identified up to September 2019 in a systematic review by Wang et al (5), and we will update their search to include all others published up to July 2022…

… For each eligible record, we will obtain relevant supplementary materials that are attached or referred to in the publication, e.g. statistical analysis plan, data management plan, PROSPERO registration record...

…We will use a standardised data extraction form which will be piloted by five independent reviewers and revised accordingly prior to commencing full extraction.” (p.7-8)

Page 9. The authors provided example of basic demographic information to be collected for representativeness: if gender is included, I think it’s worth mentioning in the list of examples.

**Response:** We have added gender as a demographic variable for collection (p.10).

Page 12. Patient and public involvement (PPI): I understand the explanation the authors, but I think PPI is necessary and they should try find and include them either in the panelist group or in the steering group.

**Response:** Thank you for this suggestion. We have invited a patient representative to participate in this project. This is articulated in the revised manuscript as follows:

“A patient representative with previous research experience will also be separately consulted to provide feedback on the list of items.” (p.9)

“Patient and public involvement

As part of the scoping review, a patient representative with previous research experience will be consulted to provide feedback on the list of items.” (p.14)

Figures: please improve their resolution

**Response:** The resolution of both figures has been improved to 300 dpi.
